# Accumulation of DNA Damage in Hematopoietic Stem and Progenitor Cells during Human Aging

**DOI:** 10.1371/journal.pone.0017487

**Published:** 2011-03-07

**Authors:** Claudia E. Rübe, Andreas Fricke, Thomas A. Widmann, Tobias Fürst, Henning Madry, Michael Pfreundschuh, Christian Rübe

**Affiliations:** 1 Department of Radiation Oncology, Saarland University, Homburg/Saar, Germany; 2 Department of Internal Medicine I, Saarland University, Homburg/Saar, Germany; 3 Department of Orthopaedic Surgery, Saarland University, Homburg/Saar, Germany; Oregon State University, United States of America

## Abstract

**Background:**

Accumulation of DNA damage leading to adult stem cell exhaustion has been proposed to be a principal mechanism of aging. Here we tested this hypothesis in healthy individuals of different ages by examining unrepaired DNA double-strand breaks (DSBs) in hematopoietic stem/progenitor cells matured in their physiological microenvironment.

**Methodology/Principal Findings:**

To asses DNA damage accumulation and repair capacities, γH2AX-foci were examined before and after exposure to ionizing irradiation. Analyzing CD34+ and CD34− stem/progenitor cells we observed an increase of endogenous γH2AX-foci levels with advancing donor age, associated with an age-related decline in telomere length. Using combined immunofluorescence and telomere-fluorescence *in-situ* hybridization we show that γH2AX-foci co-localize consistently with other repair factors such as pATM, MDC1 and 53BP1, but not significantly with telomeres, strongly supporting the telomere-independent origin for the majority of foci. The highest inter-individual variations for non-telomeric DNA damage were observed in middle-aged donors, whereas the individual DSB repair capacity appears to determine the extent of DNA damage accrual. However, analyzing different stem/progenitor subpopulations obtained from healthy elderly (>70 years), we observed an only modest increase in DNA damage accrual, most pronounced in the primitive CD34+CD38−-enriched subfraction, but sustained DNA repair efficiencies, suggesting that healthy lifestyle may slow down the natural aging process.

**Conclusions/Significance:**

Based on these findings we conclude that age-related non-telomeric DNA damage accrual accompanies physiological stem cell aging in humans. Moreover, aging may alter the functional capacity of human stem cells to repair DSBs, thereby deteriorating an important genome protection mechanism leading to exceeding DNA damage accumulation. However, the great inter-individual variations in middle-aged individuals suggest that additional cell-intrinsic mechanisms and/or extrinsic factors contribute to the age-associated DNA damage accumulation.

## Introduction

Long-lived multicellular organisms depend on tissue replenishment from small pools of tissue-specific stem cells that have the ability to self-renew and to generate differentiated progeny. A diminished capacity to maintain tissue homeostasis is a central physiological characteristic of aging, and the depletion of stem cell reserves and/or diminished stem cell functions have been postulated to contribute to aging. Recent studies suggest that accumulated DNA damage could be a principal mechanism underlying age-dependent stem cell decline.

The most deleterious form of DNA damage are DNA double-strand breaks (DSBs), which arise under physiological conditions predominantly from oxidative damage [1], or are caused by genotoxic insults such as ionizing radiation, and are repaired by non-homologous end-joining (NHEJ) and homologous recombination. Endogenous as well as radiation-induced DSBs provoke a DNA damage response characterized by the activation and recruitment of ATM (Ataxia Telangiectasia mutated) leading to the phosphorylation of Ser-139 of histone H2AX molecules adjacent to break sites. This phosphorylated form of H2AX, termed γH2AX, can be visualized by immunofluorescence analysis and form discrete nuclear foci, which co-localize with other repair factors such MDC1 (mediator of DNA damage checkpoint protein 1) or 53BP1 (p53 binding protein 1) and reflect sites of DSBs [2], [3]. γH2AX-foci analysis is a highly sensitive technique to detect DSBs, and this type of analysis has shown that the kinetics of γH2AX-foci loss strongly correlate with the time course of DSB repair [3]–[6].

Unrepaired DSBs labeled by the γH2AX-foci approach have been shown to accumulate in aging fibroblasts in culture as well as in normal tissues of aged animals [7]–[10]. Further experimental data indicate that the accumulation of DNA damage may contribute to the dysfunction of hematopoietic stem cells (HSCs) [11]. To examine the consequences of DNA damage accrual on stem cell biology, HSC reserves and functional capacities were quantified during aging of mice bearing mutations in several DNA repair pathways including nucleotide excision repair (Xpd^TTD^ mice) and NHEJ (Lig4^−/−^ and Ku80^−/−^ mice). Although deficiencies in these pathways did only partly deplete stem cell reserves with age, stem cell functional capacities were severely affected under conditions of stress, leading to loss of reconstitution and proliferative potential, diminished self-renewal, increased apoptosis and, ultimately, functional exhaustion [12], [13]. Moreover, recent studies analyzing kinetics of γH2AX-foci formation and recruitment of DSB repair proteins to the break sites in young and senescing fibroblasts indicate that the ability of human cells to sense and repair DNA damages may decline with age [14]. Together, these findings suggest that the exhaustion of stem cell function may result from the accumulation of DNA damage.

Another important aspect in this context is the telomere dysfunction in response to critical telomere shortening, which may induce DNA damage responses [15]. In primary human cells, telomeres shorten with each round of cell division, primarily due to the end-replication problem of DNA polymerase. To counteract telomere loss, a specific enzyme telomerase is required to add DNA sequence repeats (TTAGGG) to the 3′end DNA strands. Telomerase activity is important to remain the self-renewal of stem cells. However, telomeres shorten in human HSCs during aging suggesting that the level of telomerase activity is not sufficient to maintain telomere length in aging stem cells [16]. When telomeres reach critically short length, they cannot maintain proper structure at chromosomal ends, which then become unprotected. Upon telomere uncapping, the open chromosomal ends activate DNA damage responses inducing a permanent cell cycle arrest, which is referred as “replicative senescence” [17]. Thus it is conceivable that human HSCs experience telomere shortening and the induction of checkpoints in response to telomere dysfunction could contribute to the decline in stem function during aging.

However, much of the evidence implicating DNA damage accrual and telomere dysfunction as important mechanisms governing the aging process has emerged through *in-vitro* studies of human fibroblasts, or animal models with engineered mutations in diverse DNA repair pathways. Thus, the question of whether or not DNA damage accumulates in tissue stem cells of human individuals during physiological aging remains at issue. HSCs are the best-characterized stem cells in humans, functioning to ensure the lifelong production of all the diverse cell types of the hematopoietic system. HSCs have emerged as a model system for studying the effects of aging on stem cell biology since they can be purified to near homogeneity based on their unique pattern of surface markers [18]. The enrichment of CD34+ cells is current practice in hematopoietic stem cell transplantation and to date one of the most efficient cell separation methods for autologous and allogenic bone marrow transplantations [19]. However, these CD34+ cells are a heterogeneous mixture of cells that can be further separated into the immature or pluripotent CD38− cells and the more mature or committed CD38+ cells. It is postulated that immature CD34+CD38− cells could be responsible for long-term hematopoietic reconstitution, whereas more mature CD34+CD38+ cells could be responsible for early engraftment after bone-marrow transplantation [20].

Using the highly sensitive γH2AX-foci approach, we analyzed spontaneous DSBs in human HSCs and their downstream progeny of healthy individuals to evaluate endogenous DNA damage accumulation in connection with telomere shortening during physiological aging. Moreover, the formation and loss of γH2AX-foci in different stem and progenitor populations exposed to ionizing irradiation was analyzed to gain insights into age-dependent changes in DSB repair capacity. The primary objectives of this study were to evaluate whether DNA damage accrual may be a pathophysiological mechanism of stem cell aging in humans that may contribute to the diminished functional capacity of aged stem cells to maintain tissue homeostasis.

## Materials and Methods

### Ethics statement

This study (“Altersabhängige DNA Reparatur in hämatopoetischen Stammzellen” Kenn-Nr: 128/09) was approved by the local ethics committee (“Ethikkommission der Ärztekammer des Saarlandes”) and all participants gave written informed consent.

### Healthy donors and cell separation

Bone marrow and growth-factor mobilized peripheral blood of healthy volunteers (16–83 years; n = 68) or umbilical cord blood (n = 34) were obtained according to the guidelines of the local ethics committee. Mononuclear cells were separated by Ficoll gradient centrifugation (Percoll, PAA, Germany). HSCs were purified by CD34+ immunomagnetic beads (CD34 MicroBeadKit, Miltenyi, Bergisch Gladbach, Germany), and purity generally exceeded 95%. In a second separation step, the CD34+ cell subsets were magnetically separated based on CD38 (CD34 MultiSort Kit, CD38 MicroBeadKit, Miltenyi, Bergisch Gladbach, Germany) to isolate the distinct CD34+CD38+ and CD34+CD38− subpopulations.

### Irradiation

Purified cell populations diluted in RPMI 1640 (Biochrom, Berlin, Germany) were irradiated with 1 Gy or 2 Gy, respectively (90 kV; 25 mA; dose-rate: 1.2 Gy/min; 2 mm aluminum filter). At defined time-points (0.25 h; 0.5 h; 2 h; 4 h; 8 h; 24 h) after irradiation samples were centrifuged and spotted onto cover slips.

### Immunofluorescence analysis

Cells were fixed in methanol, permeabilized in acetone and incubated with anti-γH2AX-antibody (Millipore, Billerica MA, USA), anti-pATM (phosphoS1981) (Abcam, Cambridge MA, USA), anti-MDC1 or anti-53BP1 (Bethyl, Montgomery TX, USA) followed by Alexa-Fluor-488 or Alexa-Fluor-568 conjugated secondary antibodies (Invitrogen, San Diego CA, USA). Finally, samples were mounted by using vectashield mounting medium with 4′,6-diamidino-2-phenylindole (Vector Laboratories, Burlingame CA, USA). For quantitative analysis, foci were counted per cell using Nikon E600 epifluorescence microscope with 60× and 100× magnification objective (Nikon, Tokyo, Japan). Under blinded conditions, cell/foci counting were performed until at least 40 cells and 40 foci were registered in every sample.

### Telomere length analysis

Telomere length measurements were done by flow fluorescence in-situ hybridization (FISH), as described previously [16], [21], [22]. In brief, cells were incubated with FITC-labelled telomere-specific (CCCTAA)_3_ peptide nucleic acid probe (Applied Biosystems, Langen, Germany) and DNA was counterstained with LDS 751 (Exciton, Dayton OH, USA). Flow cytometric analysis was performed on FACS Calibur flow cytometer (Becton Dickinson, Heidelberg, Germany). Telomere length was expressed as mean fluorescent signal intensity. It was converted into base pairs according to a previously established standard curve [16].

### Co-localization of γH2AX-foci with telomere-specific signals

Telomere labeling was accomplished with a telomere-specific peptide nucleic acid probe conjugated with FAM-fluorochrome (TelC-FAM, Panagene, Daejeon, Korea) according to the manufacturer's protocol. Subsequently, samples were stained for γH2AX, as already described. For the different cell populations, approximately 200 γH2AX-foci were examined for co-localization with the telomere-specific signals using Nikon E600 epifluorescence microscope.

### Statistics

Linear regression analyses and Spearman's rank correlation coefficients were calculated for pre-existing as well as residual DSBs versus donor age and telomere length. To evaluate potential differences in DSB accumulation and DSB repair capacity between the diverse age-groups, statistical comparisons were performed by Mann-Whitney-U test. The criterion for statistical significance was p≤0.05.

## Results

**Figure 1:**
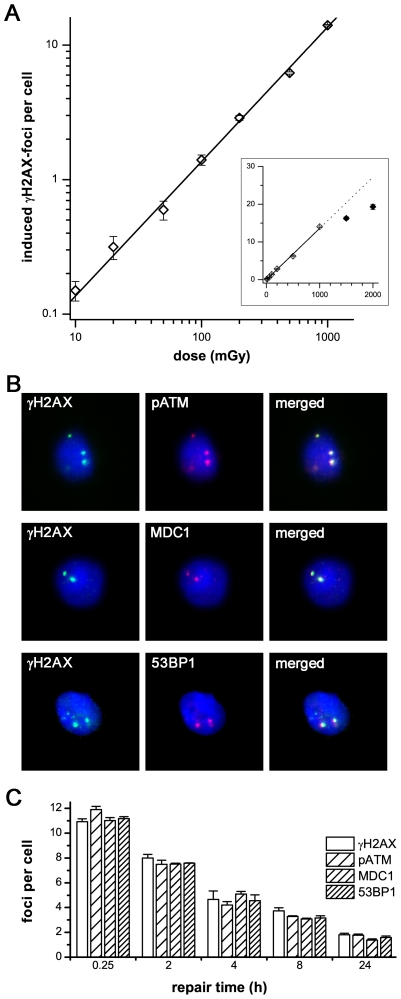
γH2AX-foci in hematopoietic stem and progenitor cells. **A**: γH2AX-foci in CD34+ cells were enumerated at 0.5 h after irradiation with doses from 10 mGy to 2000 mGy and plotted against the irradiation dose. The induction of γH2AX-foci is clearly dependent on the irradiation dose, with a linear correlation from 10 mGy to 1000 mGy. At foci levels of more than 15 foci per cell, the clustering of foci impedes their clear discrimination, leading to an underestimation of actual foci numbers (inset). **B**: Immunofluorescence double-staining of γH2AX (green) combined with pATM, MDC1 or 53BP1 in CD34+ cells, 8 h after irradiation with 2 Gy. The clear co-localization with other DNA repair factors confirms that γH2AX-foci can be used to analyze DSBs. (Original magnification, ×600) **C**: Quantitative analysis of γH2AX-, pATM-, MDC1- and 53BP1-foci in CD34+ cells at different time-points after irradiation with 1 Gy. γH2AX-foci co-localize consistently with other DNA repair factors. Error bars signify the SE of three different experiments.

γH2AX-focus formation is currently the most sensitive assay to monitor DSB levels in individuals. In pilot experiments measuring foci numbers produced by low-level radiation exposure we intended to evaluate the dose range yielding linearity between foci number and dose. Figure 1A shows that the induction of DSBs is clearly dependent on the irradiation dose, with a linear correlation even after very low doses. Moreover, these γH2AX-foci consistently co-localize with other DNA repair factors such as pATM, MDC1 and 53BP1 (Fig. 1B, 1C), confirming that this marker can be used to analyze DSBs. In further experiments we could show that endogenous foci numbers do not vary during culture time, with stable foci counts over a period of more than 72 hours. Moreover, DNA flow cytometry analysis using propidium iodide revealed that CD34+ and CD34− cells obtained from umbilical cord blood and adult bone marrow show similar cell cycle distributions with about 85% in G1/G0-, 5–9% in S- and 3–7% in G2/M-phase (data not shown). Collectively, these findings suggest that γH2AX-foci analysis represent a reliable and exquisitely sensitive technique to monitor DSBs in human haematopoietic stem and progenitor cells.

**Figure 2:**
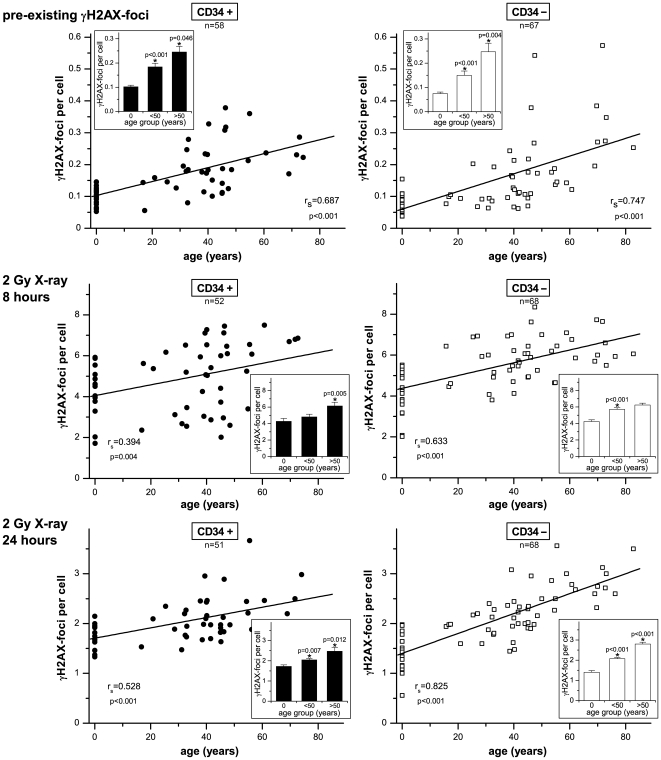
Accumulation of DSBs and declining DSB repair capacities with advancing age. The number of pre-existing γH2AX-foci (upper panels), and radiation-induced γH2AX-foci at 8 hours (middle panel) and 24 hours (lower panels) after irradiation with 2 Gy was plotted against donor age, depicted separately for CD34+ and CD34− cells. Linear regression analyses were performed (solid lines) and Spearman's rank correlation coefficients (r_s_) were calculated. For three different arbitrary groups (age: 0/<50/>50 years) mean γH2AX-foci levels are displayed in bar graphs. Error bars represent the SE within the specific group. *Statistically significant difference to the next younger age group (p<0.05).

Subsequently, we analyzed the pre-existing DSBs in CD34+ and CD34− cells derived from various stem cell sources (umbilical cord blood, growth-factor mobilized peripheral blood, bone marrow), by counting γH2AX-foci per cell. In figure 2 (upper panels) the number of γH2AX-foci was plotted against the chronological donor age, depicted separately for CD34+ and CD34− cells. In CD34+ cells obtained from umbilical cord blood (age-group 0) the number of γH2AX-foci was generally fairly low with only minor variations (0.102±0.006 foci/cell). With increasing age we observed a steady rise in the number of γH2AX-foci, with significantly elevated foci levels in the age-group of <50 years (0.184±0.015 foci/cell; p<0.001) and even higher foci levels in the age-group of >50 years (0.245±0.023 foci/cell; p = 0.046). Similar to their stem/progenitor counterpart, the more differentiated CD34− cells obtained from cord blood revealed only low γH2AX-foci levels with only minor variations (0.074±0.006 foci/cell). Moreover, γH2AX-foci analysis of CD34− cells revealed a similar increase of unrepaired DSBs with increasing donor age, with significantly elevated foci levels in the <50 (0.145±0.018 foci/cell; p≤0.001) and >50 age-groups (0.247±0.036 foci/cell; p = 0.004). These findings suggest that unrepaired DSBs accumulate continuously in hematopoietic stem/progenitor cells (CD34+) and their more differentiated progeny (CD34−) during physiological aging.

Next, we examined the CD34+ and CD34− cells for their abilities to respond to ionizing irradiation. The DSB repair capacity of each individual was analyzed by counting γH2AX-foci at defined time-points after irradiation with 2 Gy. At 0.5 h post-irradiation comparable levels of γH2AX-foci were induced in CD34+ and CD34− samples regardless of the donor age (data not shown). In figure 2, the residual DSBs (γH2AX-foci per cell) were plotted against the donor age, depicted separately for the repair-times 8 h (middle panels) and 24 h post-irradiation (lower panels). CD34+ and CD34− cells obtained from cord blood samples generally revealed quite low levels of residual γH2AX-foci at 8 h (CD34+: 4.275±0.341 foci/cell; CD34−: 4.234±0.211 foci/cell) and 24 h post-irradiation (CD34+: 1.729±0.068 foci/cell; CD34−: 1.406±0.081 foci/cell, compare insets), indicating fairly efficient DSB repair capacities. In contrast, significantly higher foci levels were registered at 8 h and particularly at 24 h post-irradiation in the CD34+ and CD34− cells of the <50 years age-group (CD34+: 2.047±0.068 foci/cell; p = 0.007; CD34−: 2.072±0.063 foci/cell; p<0.001) and >50 years age-group (CD34+: 2.474±0.186 foci/cell; p = 0.012; CD34−: 2.798±0.093 foci/cell; p<0.001, compare insets). Collectively, with increasing age we observed rising levels of residual γH2AX-foci after exposure to ionizing radiation, suggesting diminished DSB repair capacities in older individuals.

To evaluate the relationship between endogenous accumulation of unrepaired DSBs and individual DSB repair capacity, the pre-existing γH2AX-foci were plotted against the residual foci (24 h after irradiation) for every individual. As shown in Figure S1, significant correlations between the level of naturally pre-existing γH2AX-foci and residual foci after irradiation were noticeable in CD34+, and even more pronounced in CD34− cells of different-aged donors, suggesting that the individual DSB repair capacity may determine the degree of DNA damage accumulation.

**Figure 3:**
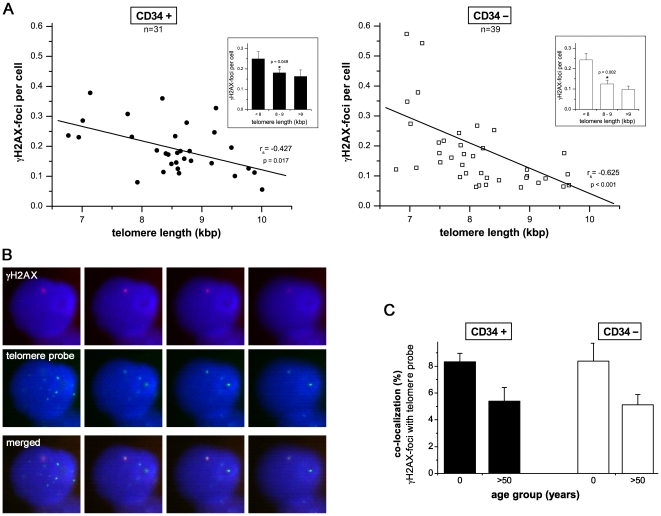
Accumulation of DSBs and progressive telomere-shortening. **A**: The number of pre-existing γH2AX-foci per cell was plotted against telomere length depicted separately for CD34+ and CD34− cells. Linear regression analyses were performed (solid lines) and Spearman's rank correlation coefficients (r_s_) were calculated. For three different arbitrary groups (telomere length: <8/8–9/>9 kb) mean γH2AX-foci levels are displayed in bar graphs. Error bars represent the SE within the specific group. *Statistically significant difference to the next younger age group or next group with shorter telomere (p<0.05). **B**: Co-localization between γH2AX- and telomere-signals. Co-localization of endogenous γH2AX-foci (red) with telomere-specific DNA (green FISH signal). DAPI staining (blue) indicates DNA. (Original magnification, ×600) **C**: Quantitative analysis of the extent of co-localization between γH2AX- and telomere-signals. In CD34+ and CD34− cells obtained from cord blood and bone marrow of aged individuals (>50 years), respectively, approximately 200 γH2AX-foci were screened per data point in independent experiments. Error bars signify the SE of five different experiments.

Previous studies have shown that the measurement of telomere length can give valuable insight into the replicative history of cells, indicating their biological age [23]. In the present study, we correlated the telomere length and number of γH2AX-foci per cell for CD34+ and CD34− cells. CD34+ and CD34− cells obtained from umbilical cord blood revealed long telomeres (>9 kb) and low γH2AX-foci levels for CD34+ (0.164±0.031 foci/cell) and CD34− cells (0.097±0.017 foci/cell) (Fig. 3, upper panels). Moreover, analyzing CD34+ and CD34− cells of healthy adults we were able to confirm that telomere length shortens continuously with age (Figure S2). This highly significant age-related decline in telomere length was associated with increasing foci levels in both CD34+ (8–9 kb: 0.181±0.016 foci/cell; ≤8 kb: 0.250±0.035 foci/cell; p = 0.049) and CD34− cells (8–9 kb: 0.125±0.018 foci/cell; ≤8 kb: 0.243±0.032 foci/cell; p = 0.002, compare Fig. 3A insets). However, the Spearman's correlation coefficients (r_s_) describing the relationship between γH2AX-foci per cell and donor age (Fig. 2, upper panels) or telomere length (Fig. 3A), respectively, were clearly higher for the chronological age.

Since telomere dysfunction in response to critical telomere shortening may induce DNA damage responses, we assessed the extent of co-localization between the γH2AX-foci and telomeres by the combined immunofluorescence and telomere-fluorescence *in-situ* hybridization (Fig. 3B; Figure S3). In CD34+ and CD34− cells obtained from cord blood and bone marrow of aged individuals (>50 years) between 5–8% of the γH2AX-signals coincided with telomere signals, whereas the frequencies of co-localization were even slightly higher in cord blood derived HSCs (Fig. 3C). Although these results indicate that some of the γH2AX-foci are localized at or near telomeres, our findings do not support the notion that progressive telomere shortening contributes to the accumulation of γH2AX-foci in physiologically matured HSCs.

**Figure 4:**
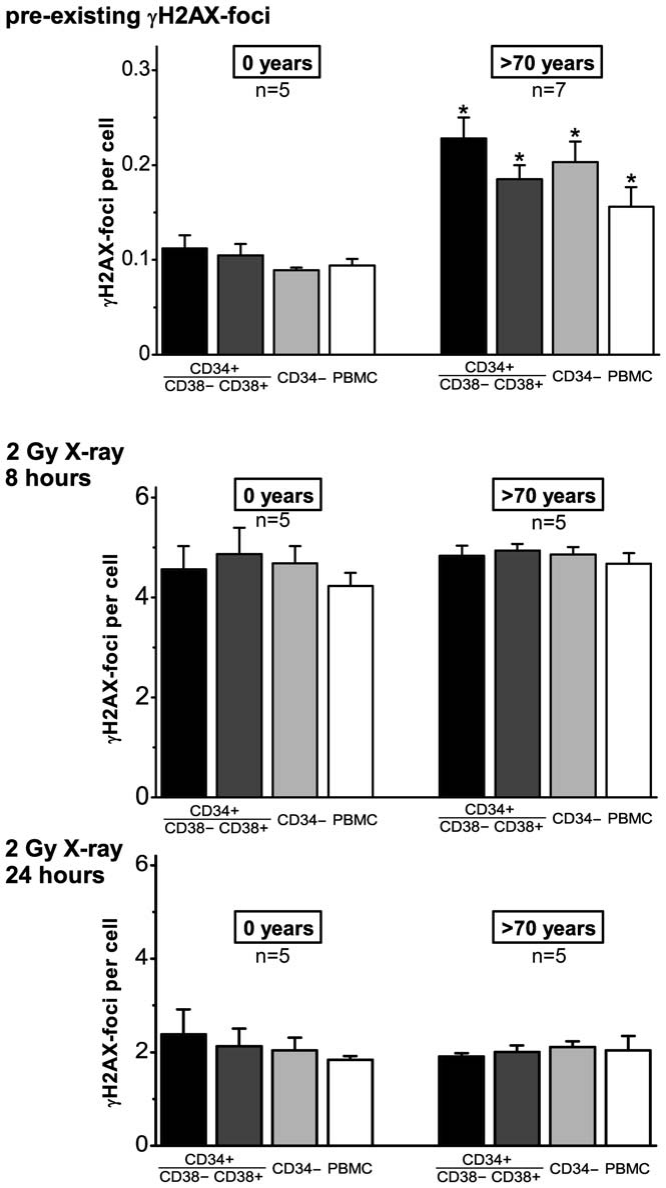
Accumulation of DSBs in the different stem/progenitor populations during healthy aging. The number of pre-existing γH2AX-foci (upper panel), and radiation-induced γH2AX-foci at 8 hours (middle panel) and 24 hours (lower panel) after irradiation with 2 Gy was counted in CD34+CD38−, CD34+CD38+, CD34− and PBMC cells derived from cord blood (0 years) and bone marrow of healthy elderly (>70 years). Error bars represent the SE within the specific group. *Statistically significant difference to the corresponding cord-blood derived stem/progenitor population (p<0.05).

To explore the DNA damage accrual in different stem and progenitor cell populations during healthy aging, we screened for bone marrow samples of elderly individuals (>70 years) characterized by a good health status, notably without any chronic diseases. In subsequent experiments we analyzed the pre-existing γH2AX-foci in CD34+CD38−, CD34+CD38+ and CD34− cells derived from bone marrow of these elderly donors and compared their DNA damage accrual with the corresponding cell populations obtained from umbilical cord blood. In all cell fractions, we observed significantly increased numbers of γH2AX-foci for these elderly donors, with up to twice as high foci levels compared to the cord blood derived subpopulations (Fig. 4, upper panel). Long-lived CD34+CD38− cells revealed the highest level of γH2AX-foci (>70 years: 0.228±0.022 foci/cell; 0 years: 0.112±0.014 foci/cell, p = 0.005), maybe reflecting the continuous accumulation of DNA damage during their extended lifespan. But also more committed progenitor cells (CD34+CD38− and CD34−) and even peripheral blood mononuclear cells (PBMCs) revealed elevated foci levels compared to the cord blood derived subtypes (Fig. 4, upper panel). These findings indicate that these different subtypes of hematopoietic stem and progenitor cells accumulate persistent DNA lesions that may contain unrepairable DSBs.

In subsequent experiments, we quantified γH2AX-foci at 8 h and 24 h after irradiation with 2 Gy, to find out whether genotoxic insults such as ionizing radiation differently affect these various subpopulations. Significantly, we observed a nearly identical decline of γH2AX-foci levels in CD34+CD38−, CD34+CD38+ and CD34− cells after exposure to irradiation, suggesting similar efficiencies of DSB processing in all analyzed cell fractions (Fig. 4, middle and lower panel). Unexpectedly, the DSB repair capacities of these different cell populations derived from matured bone marrow and umbilical cord blood were equally efficient, irrespective of their wide difference in donor age. At first glance, these results appear to be contradictory to our previous findings (compare Fig. 2), showing evidence for a decline of DNA repair with age. However, these discrepancies can be explained by our strict donor selection, suggesting that the observed effectiveness of DNA repair in these elderly individuals may be related to their excellent health status.

## Discussion

Accumulation of DNA damage leading to adult stem cell exhaustion has been proposed to be a principal mechanism of aging. Here, we tested this hypothesis in healthy individuals by examining unrepaired DNA damage in hematopoietic stem/progenitor cells aged in their natural environment under physiological conditions. Analyzing CD34+ and CD34− stem/progenitor cells we observed an increase of endogenous γH2AX-foci levels with advancing donor age, associated with an age-related decline in telomere length. Using combined immunofluorescence and telomere fluorescence *in-situ* hybridization, we show that the age-dependent increase of DNA damage involve predominantly non-telomere-associated γH2AX-foci, arguing against the hypothesis that telomere dysfunction in response to critical telomere shortening plays a primary role in producing senescence- and age-associated DNA damage in humans. The highest inter-individual variations for non-telomeric DNA damage were observed in middle-aged donors, whereas the individual DSB repair capacity appears to determine the extent of DNA damage accrual. However, analyzing different stem/progenitor subpopulations obtained from healthy elderly (>70 years), we observed an only modest increase in DNA damage accrual, most pronounced in the primitive CD34+CD38− enriched subfraction, but sustained DNA repair efficiencies, suggesting that healthy lifestyle may slow down the natural aging process. Based on these findings we conclude that age-related non-telomeric DNA damage accrual accompanies physiological stem cell aging in humans. Moreover, aging may alter the functional capacity of human stem cells to repair DSBs, thereby deteriorating an important genome protection mechanism leading to exceeding DNA damage accumulation. However, the great inter-individual variations in middle-aged individuals suggest that additional cell-intrinsic mechanisms and/or extrinsic factors contribute to the age-associated DNA damage accumulation.

There is growing evidence that stem cell aging is not purely an intrinsic process, but is also regulated by external stimuli within specific microenvironments, the so called “stem cell niche”. The stem cell niche governs asymmetric cell division of two daughter cells that may either self-renew to replenish the stem cell pool or to differentiate to more committed cells. Furthermore, anchorage of HSCs to specific niches in the bone marrow appears to support and protect quiescent stem cells from damage and stress induced exhaustion [24]. Recent results provide evidence, that the supportive function of the niche is modified during the process of aging, thereby affecting the extrinsic mechanisms of stem cell control [25].

The integrity of the genome can be affected by a variety of environmental and lifestyle factors which may contribute to the impairments in organ maintenance and function during human aging. Recent studies indicate that lifestyle factors (such as nutrition, physical exercise, tobacco smoking, alcohol consumption) have an age-independent influence on the accumulation of DNA damage and telomere dysfunction in human blood [26]. Moreover, there are emerging reports that lifestyle interventions can reduce the levels of DNA damage and telomere shortening *in-vivo* [27]–[29].

In the present study, we observed an age-dependent accumulation of DSBs and decline in DSB repair functions in human HSCs aged in their physiological microenvironment. Thus, DNA damage accrual is not a response to artificial cell cultures conditions (as previously discussed in aging fibroblasts), but may be part of both intrinsic and extrinsic mechanisms governing the physiological aging process in human stem cells. HSCs obtained from umbilical cord blood exhibited low levels of endogenous DNA damage and highly efficient DNA repair capacities with only minor inter-individual variations (Fig. 2). In contrast, HSCs of middle-aged (<50 years) and even more pronounced of aged donors (>50 years) revealed clearly higher amounts of unrepaired DNA damage and less efficient repair capacities, with broader variations in both DSB accumulation and repair capacities (Fig. 2). These results suggest that inter-individual differences in DSB accumulation and repair become more apparent with increasing age, maybe reflecting different lifestyle exposures (such as smoking or obesity) that may contribute to aging. However, analyzing different stem and progenitor subpopulations obtained from healthy elderly (>70 years), we observed only modest increase in DNA damage accrual and sustained DNA repair efficiencies, suggesting that healthy lifestyle may slow down the natural aging process. In actual fact, our exploration of healthy elderly implies a strict selection as only individuals reaching an advanced age in good health and without any chronic diseases were included in this study. Thus, analyzing healthy elderly the whole spectrum of inter-individual differences in age-dependent DNA damage accumulation and repair is certainly not covered in this clinical study.

In most mature cells, eroding telomeres act as an internal clock that limits the number of divisions that a cell can perform [23]. In contrast, stem cells often express telomerase which can elongate telomeres and extend their replicative potential [15]. In line with former studies, we could previously show that the CD34+ cell populations from healthy donors uniformly express only baseline telomerase activity, not sufficient to maintain telomere length during aging [16], [22]. In the present study we could confirm an age-related shortening of telomeres in both CD34+ and CD34− cells (Figure S1), associated with increasing levels of unrepaired DNA damage (Figure 3, upper panel). Oxidative stress has been shown to be a major causal factor for telomeric DNA damage contributing to telomere attrition [30]. Thus, it appears likely that telomere damage leading to telomere-shortening is functionally interrelated with endogenous DSBs, probably both induced by oxidative stress [31]. However, plotting the endogenous γH2AX-foci against the telomere length (instead of chronological donor age) revealed lower correlations coefficients in CD34+ and CD34− cells (Fig. 3A), indicating that the age-related increase of DNA damage may derive from telomere-independent mechanisms. Moreover, we assessed the extent of co-localization between γH2AX-foci and telomeres in CD34+ and CD34− cells obtained from cord blood and bone marrow of aged donors. Irrespective of the donor age only a low percentage of the γH2AX-signals coincided with telomere signals, strongly supporting a non-telomeric origin for the majority of γH2AX-foci (Fig. 3B, C; Figure S3).

In our study we observed a significant correlation between the amount of unrepaired DSBs and the DSB repair capacity (Fig. 2, Figure S1), suggesting that the DSB repair itself may change with age and that the age-associated increase of unrepaired DSBs may reflect the reduced efficiency of NHEJ, leading to slower processing of DSBs. In addition, NHEJ cannot accurately reconstitute sequence information lost at DSBs, and thus may result in mutational changes at the site of repair [32]. Previous work finds evidence that the efficiency and fidelity of NHEJ declines during cellular senescence in human fibroblasts, and therefore DSB resolution becomes clearly more error-prone with age [33]. Based on our data we suggest that this age- and probably lifestyle-related decline in DSB repair may lead to a self-amplifying cycle with delayed kinetics in DSB processing reflecting a decreased efficiency and fidelity of repair processes leading to the accumulation of mutations. Accrual of genomic damage in turn may cause a gradual decline of the functional capacity to repair DNA damage, thereby contributing to genomic instability and, ultimately, to stem cell exhaustion.

Many proteins involved in the DNA damage response aggregate as microscopically visible foci at sites of DSBs after exposure to ionizing radiation, and it is generally assumed that persisting γH2AX-foci may indicate the presence of unrepaired or misrepaired DSBs. However, our understanding of the biological relevance of residual DNA repair foci still remains limited. Recent experimental findings suggest that persisting γH2AX-foci may reflect chromatin alterations after DNA rejoining rather than unrepaired DSBs [34]. DSB repair is a highly coordinated process that requires the unraveling of the compacted chromatin structure to facilitate repair machinery access and then restoration of the original undamaged chromatin state. Incomplete or incorrect restoration of chromatin structure can leave a DSB-induced epigenetic memory of damage with potentially pathological repercussions. Considering the critical importance of chromatin organization to correct gene expression control, proper restoration of epigenetic patterns following DSB damage would be crucial to avoiding perturbation of transcriptional programs, involving the aberrant activation or silencing of genes. Moreover, preservation of transcriptional states essential to cell identity and function requires the correct, high-fidelity propagation of the underlying pattern of epigenetic marks from mother to daughter cell. It is therefore conceivable that our finding of similar endogenous γH2AX-foci levels in different stem and progenitor cells derived from the same individual may reflect the faithfully reproduced epigenetic information in the event of damage, resulting in chromatin alterations that are transmissible over multiple cell generations. Another important point in this context is the varying chromatin architecture in the course of lineage-specific differentiation. Lineage-restricted cells generally have more condensed chromatin architecture than pluripotent stem cells which employ unique histone modifications to regulate the balance between pluripotency and differentiation [35]. Recent studies have shown that pluripotent mouse embryonic stem cells exhibit excessive numbers of γH2AX-foci in the absence of measurable DSBs, which appear to be associated with global chromatin decondensation rather than pre-existing DNA damage [36]. However, analyzing the formation and removal of γH2AX-foci in the different stem and progenitor cells (CD34+CD38−, CD34+CD38+ and CD34− subpopulations) we observed no differences in the induction and repair kinetics, suggesting that potential differences in their chromatin organization seem to have no impact on sensing and processing of radiation-induced DNA damage.

Advancing age is accompanied by a number of pathophysiological changes in the hematopoietic system, whose etiology suggests loss of homeostatic control and possible stem/progenitor cell involvement. The most clinically significant of these changes are the decreased competence of the adaptive immune system [37], [38], and the increased incidence of leukemia and lymphoma [39]. Due to the low number of available cells in our clinical study, we could not examine whether or not aging affects the number and/or function of human HSCs. However, clinical data indicate that advanced donor age is a strong negative prognostic marker for survival in allogenic bone marrow transplantations, which might indicate impaired function of HSCs from aged donors [40].

### Conclusions

Our study provides compelling evidence that the accumulation of DNA damage and the reduced ability to repair DNA damage plays an important role in the physiological aging of humans. Given the close association between aging and cancer development, the relevance of these observations has wider implications. Only tissue stem cells possess the proliferative capacity and lifespan necessary to accumulate genetic damage, and are therefore considered to be the principal target for transforming mutations leading to cancer. Decreasing efficiencies in DSB repair may contribute to genomic instability in tissue stem cells and successive mutagenic events may result in both gradual decline of stem cell functions and tumor formation. Increased incidence of cancer with age is the best-known evidence of age-related genomic instability. A better understanding of the underlying mechanisms governing the aging process could ultimately lead to the development of molecular strategies aiming to treat age-related degenerative and malignant diseases.

## Supporting Information

Figure S1 **The individual DSB repair capacity determines the degree of endogenous DNA damage accumulation.** Pre-existing γH2AX-foci were plotted against the residual foci (24 h after irradiation with 2 Gy) for every individual, depicted separately for CD34+ and CD34− cells. Linear regression analyses were performed (solid lines) and Spearman's rank correlation coefficients (r_s_) were calculated.
(TIF)

Figure S2 **Age-related telomere shortening.** Telomere lengths were plotted against the donor age, depicted separately for CD34+ and CD34− cells. Linear regression analyses were performed (solid lines) and Spearman's rank correlation coefficients were calculated.
(TIF)
