# Supplementary figures and images for: Accumulation of DNA Damage in Hematopoietic Stem and Progenitor Cells during Human Aging
Source: PLoS One. 2011 Mar 7;6(3):e17487. doi: 10.1371/journal.pone.0017487 (PMC3049780; doi:10.1371/journal.pone.0017487)

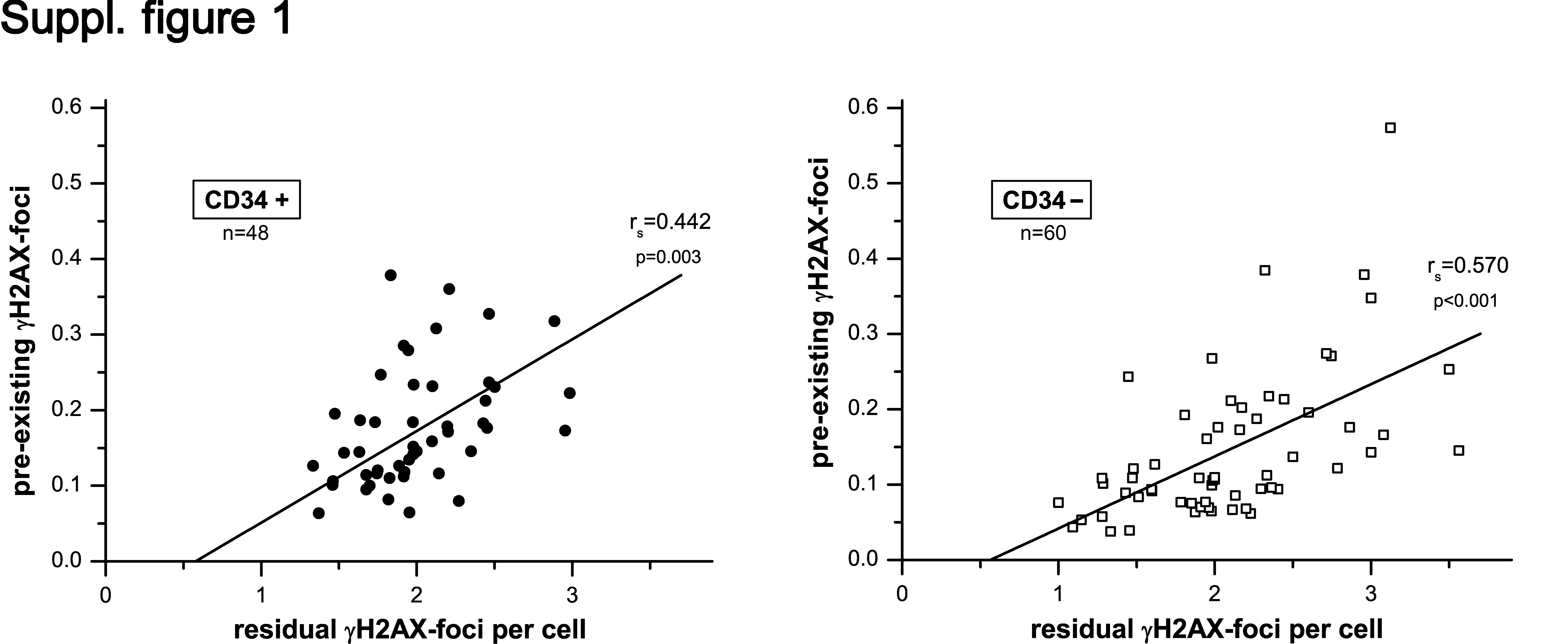

Supplement: Figure S1 — The individual DSB repair capacity determines the degree of endogenous DNA damage accumulation. Pre-existing γH2AX-foci were plotted against the residual foci (24 h after irradiation with 2 Gy) for every individual, depicted separately for CD34+ and CD34− cells. Linear regression analyses were performed (solid lines) and Spearman's rank correlation coefficients (rs) were calculated. (TIF) [file pone.0017487.s001.tif]

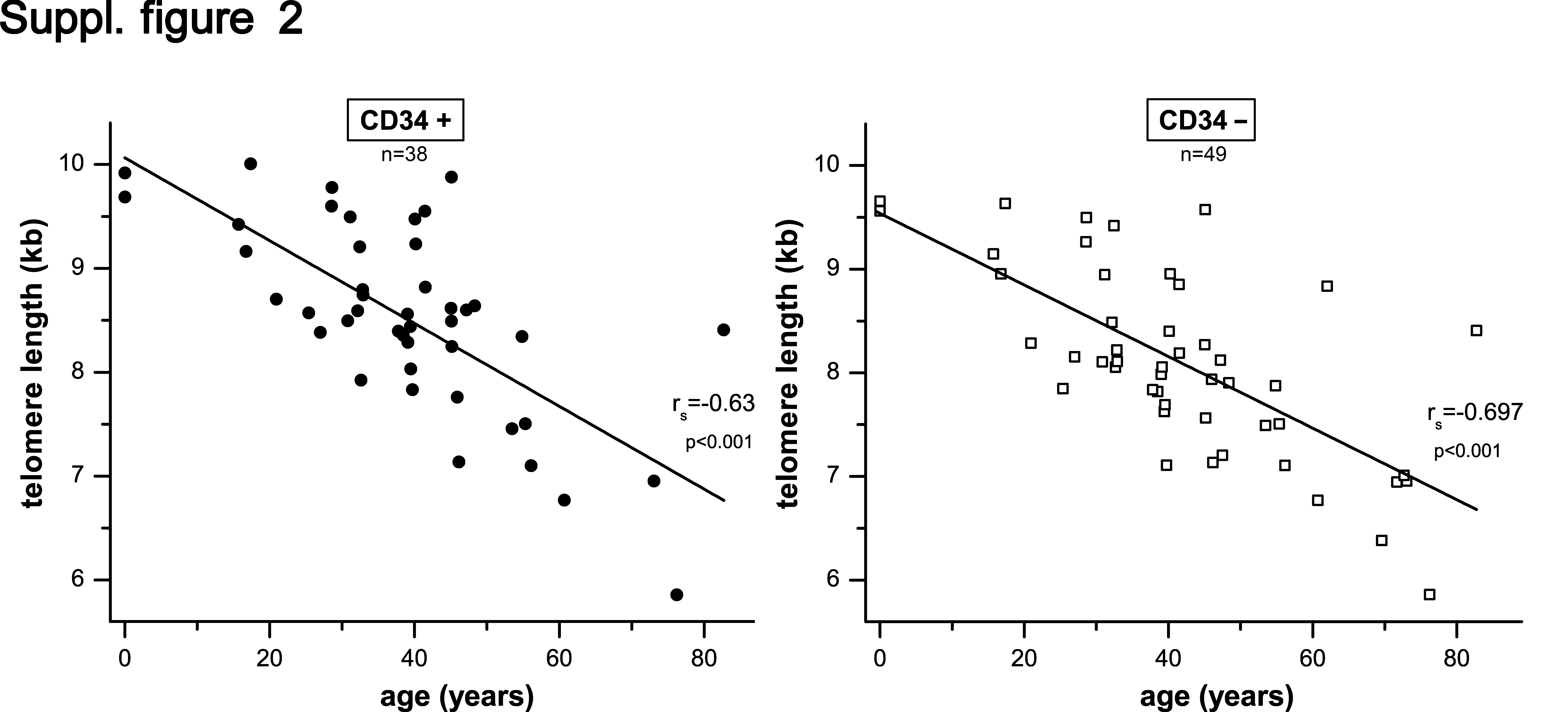

Supplement: Figure S2 — Age-related telomere shortening. Telomere lengths were plotted against the donor age, depicted separately for CD34+ and CD34− cells. Linear regression analyses were performed (solid lines) and Spearman's rank correlation coefficients were calculated. (TIF) [file pone.0017487.s002.tif]
